# Community satisfaction with the process of curative care administered by community health workers in the Boussé and Boussouma health districts in Burkina Faso

**DOI:** 10.1371/journal.pgph.0003951

**Published:** 2025-05-08

**Authors:** Hamed Sidwaya Ouédraogo, Ahmed Kaboré, Abdoul-Guaniyi Sawadogo, Badra Ali Traoré, Mamadou Traoré, Massoudou Harouna Maiga, Maxime Koiné Drabo

**Affiliations:** 1 Ministry of Health and Public Hygiene, Ouagadougou, Burkina Faso; 2 Public Health Laboratory of the UFR/SDS, Doctoral School of Health Sciences (ED-2S), Joseph KI-ZERBO University, Ouagadougou, Burkina Faso; 3 Coordination of the Master of Public Health UFR/SDS, Joseph KI-ZERBO University, Ouagadougou, Burkina Faso; 4 Institute of Sports Sciences and Human Development (ISSDH), Joseph KI-ZERBO University, Ouagadougou, Burkina Faso; 5 Jhpiego Corporation, Burkina Faso Office, Ouagadougou, Burkina Faso; 6 Independent consultant in local development, Ouagadougou, Burkina Faso; 7 Institute for Research in Health Sciences (IRSS)/CNRST, Ouagadougou, Burkina Faso; PLOS: Public Library of Science, UNITED STATES OF AMERICA

## Abstract

The development of accessible care for communities is a priority in Burkina Faso. To achieve this, the country has developed integrated community management of childhood illnesses following the global agenda, involving trained and institutionalized community health workers and free community care. This study aims to identify factors likely to influence the quality of the process of this community management. We have undertaken to conduct a mixed cross-sectional study with a descriptive and analytical aim. The data were collected in the health districts of Boussé and Boussouma from February 20 to March 30, 2023 (quantitative data) and from January 12–30, 2024 (qualitative data) using a questionnaire and an interview grid, then the data were analyzed with SPSS IBM 25 and Nvivo 14. At the end of this study, households reported benefiting from curative from community health workers, but don’t use it first in some cases for fear of wasting time and also on the advice of these community health workers. Binary logistic regression analysis identified factors influencing the quality of the process of this community care such as the ease of contacting the community health workers, the level of education of households and courtesy of the community health workers during care. In summary, households trust the integrated community management of childhood illnesses. Their satisfaction with this strategy could be improved by strengthening contacts between communities and community health workers and their technical and communication capacities.

## 1. Introduction

The provision of integrated health care close to communities is an international priority. In particular, the global community faces an urgent need to strengthen basic health care systems to ensure better health coverage for women and children [1]. Several reflections have fed the global health agenda, particularly in the 1980s, for strengthening access to primary health care [2]. The inadequate results in progress towards the Millennium Development Goals (MDGs) have reinforced positioning primary health care among the flagship strategies promoted by the World Health Organization (WHO). they have precipitated decision-making and defined perspectives focused on they have precipitated decision-making and defined perspectives focused on the achievement of universal health coverage through frontline health workers trained and deployed to provide essential services to rural populations especially in the WHO African region [3]. The development of primary health care (PHC) [4], which was struggling to be implemented due to the inadequacy of healthcare human resources [5], has found a second wind through community health workers (CHWs), who were defined as volunteers recruited from the community, trained and equipped to serve as health auxiliaries in the service of their communities [3,5,6]. These community health workers (CHWs) were to be the interface between the community and health teams [7,8]. Their curative services mainly targeted the most deadly childhood diseases such as malaria, pneumonia and diarrhoea [6,9]. Their curative services are based mainly on the integrated strategy for community management of childhood diseases jointly supported by WHO and the United Nations Children’s Fund (UNICEF) [6] and implemented to strengthen geographic and financial access to care for populations [6,10–12]; a real bridge to progress towards universal health coverage [13,14]. Burkina Faso has implemented integrated community management of childhood illnesses (iCCM) since the revolutionary years of 1983 [15] and continued to this day, like several countries in sub-Saharan Africa [11,16–18]. It is one of the major priorities for Burkina Faso in the fight against infant morbidity and mortality [19,20] and the adaptation of the health system [21].

The results obtained in the implementation of this community care are slow to produce the desired impacts [13,19,20,22,23]. The use of CHWs by the population has remained low according to certain studies and the coverage of the population insufficient [10,22] for very variable reasons [8,10,19].

In Burkina Faso, several studies were carried out on this issue before the implementation of major reforms by the Government for the harmonization of the CHWs profile, its financial treatment and the consideration of community services in the free healthcare policy in 2018 [24]. All this motivated the conduct of this study. It aims to research the factors influencing the quality of the iCCM process in the health districts of Boussé and Boussouma in Burkina Faso in order to guide decision-making to strengthen household satisfaction and improve the use of this local curative care.

## 2. Materials and methods

### 2.1. Ethics statement

This study received authorization from the National Health Research Ethics Committee housed within the Ministry of Health and Public Hygiene of Burkina Faso (deliberation No. 2023-03-061). Informed consent was obtained from all subjects participating in the study.

### 2.2. Type and period of study

We conducted a cross-sectional study with a descriptive and analytical aim, using a mixed approach (qualitative and quantitative methods). The data were collected in two phases; the first from February 20 to March 30, 2023 (quantitative data) and the second from January 12–30, 2024 (qualitative data).

### 2.3. Study setting

The study was conducted in the health districts of Boussouma and Boussé in Burkina Faso. The Boussé health district located in the Central Plateau region covers five (05) communes and has a population of 199999 inhabitants and has 33 health facilities. The Boussouma health district is located in the Centre-Nord region covering three municipalities and has a population of 239894 inhabitants and has 29 health facilities. These districts fall under the most operational level of the Burkinabe health system, whose overall architecture is pyramidal, going from the national level to these operational structures.

### 2.4. The population studied

The study focused on heads of households and child guardians.

### 2.5. Conceptual, technical approach and data collection tools

This study was built according to the BENEGUISSE model for analyzing the quality of care. A model with five dimensions was described: geographical accessibility, organizational, accessibility, interpersonal communication, technical competence, and continuity of care (S1 Table). We focused on access to CHWs services according to households’ opinions. This model is used because it highlights the perceptions of beneficiaries and provides health measures aimed at improving the use of services. Data were collected from households in two stages.

Quantitative data were collected using a questionnaire imported onto smartphones generated using Kobotoolbox software, tools used for the first phase of data collection. This questionnaire was pre-tested in the Central region. Interviewers were trained in one day by a collection assistant with a degree in social sciences and mastering the processes of preparing and implementing surveys before their deployment. For the second phase, the interview grid was printed and made available to the interviewers who were also trained for the collection assistant. They were equipped with dictaphones and conducted the interviews face to face.

Data were collected on variables such as: socio-demographic characteristics, availability of human resources including CHWs, types of services offered by CHWs, knowledge of the community service package, level of implementation of community activities, availability of inputs and variables related to the five components of the model we used, namely geographical accessibility, temporal accessibility, cultural accessibility, quality of the care environment, reception of the CHWs, courtesy when administering care to the child, awareness, practical advice when administering care to the child. To operationalize these variables, we used a Likert scale with the five points: “very satisfied”, “satisfied”, “fairly satisfied”, “not very satisfied”, “not at all satisfied”.

### 2.6. Sampling

The sampling was conducted in several stages. For the quantitative methodology, a two-level cluster sampling was carried out. A random draw without replacement of the villages to be surveyed was carried out from the list of villages to be surveyed in the two health districts using Excel (XLSTAT) software. Then, a census of households was carried out in each sampled village and the information was entered. The list of households to be surveyed was generated automatically and the heads of household or guardians of children were surveyed. Any household without a child under five years old was replaced.

For the qualitative part of the study, a simple random selection made it possible to choose one health facility per commune before choosing a village at least 5 km from the site of the health facility in order to comply with the criteria for choosing villages to be covered by the ICCM. We also took into account the security context that conditions the data collection team’s mobility. In each village, two focus groups were set up using an interview grid to gather their perceptions and experiences of their children’s illness episodes and the use of CHW services. The number of people in these focus groups was at least five. Given the social context, we had groups of women and groups of men from households with children under five in their households.

### 2.7. Data processing, analysis and ethical aspects

The quantitative data were exported to Excel software. Qualitative data were collected in the local language or in French, depending on the participant. Data in the local language were translated into French before being transcribed. All data were transcribed into English on Word with the help of a professional interpreter. The quantitative data (S1 Data) were cleaned and analyzed using IBM SPSS version 25 software. We used binary logistic regression with the ‘enter’ method to obtain the model. The chi-square test was used and the analyses were carried out at a 95% confidence level. This regression was intended to determine the factors influencing access to CHWs services by households when their children were sick.

The interviews were transcribed using Microsoft Word processing software and those in national languages were translated into French beforehand before transcription. We conducted discourse analysis using a descriptive approach to identify key elements of the discourse [25]. We used NVIVO 14 software for this thematic content analysis.

## 3. Results

### 3.1. Sociodemographic characteristics of respondents

Our study involved a total of 960 households who agreed to answer our questions. The median age of respondents was 34 years (16.80).

The proportion of female respondents was 78.1% (750/960). The average distance between households and the health facility was 7.29 ± 4.6 km. The majority of participants were not in education and those who were farmers by occupation were 50.7% (487/960) shown in table 1. Among these households 4.9 (47/960) were households of internally displaced persons. Qualitative data from twenty focus group sessions were considered satisfactory and used in this study. For this study, variables that are related to the access of community care provided by CHWs and were analysed the quality of this care.

**Table 1:** Breakdown by sex, education and occupation of respondents at household level.

Variables		Frequency	Percentage
**Gender**			
	Female	750	78.1
	Male	210	21.9
	Total	960	100.0
**Education level**			
	Literate	42	4.4
	No	622	64.8
	Koranic school	25	2.6
	Primary	192	20.0
	Secondary	75	7.8
	Superior	4	0.4
	Total	960	100.0
**Profession**			
	Private agent	3	0.3
	Agent du public	1	0.1
	Other	12	1,3
	Retailer	95	9,9
	Cultivator	487	50.7
	Breeder	26	2.7
	Housekeeper	336	35.0
	Total	960	100.0
**Marital status**			
	Single	45	4.7
	Divorced	9	0.9
	Married	884	92.1
	Common-law union	9	0.9
	Widow	13	1.4
	Total	960	100.0

### 3.2. Assessment of the context of the offer based on access

According to the assertions of the respondents listed in Table 2, it appears that the availability of CHWs services is effective. The majority of households reassured that CHWs are people from their communities who offer services within said communities and also expressed great satisfaction with the possibilities of curative care without having to resort to the health facilities. According to some of those interviewed, this helps to reduce the distances they have to travel to receive care.

**Table 2:** Breakdown of some quotes from focus group participants according to whether they were satisfied or dissatisfied.

Areas of the quality of care model according to Beneguissé	Illustrative quotes ranked by satisfaction	
**Argument on satisfactory quality**	**Argument on unsatisfactory quality**
**Geographic access**	“Yes they are with us” Woman_R5_FG3 “I know them, we are together here” Man_R1_FG7 “Yes there are periods during which they walk around with medicines for the children” Man_R3_FG10 “Yes since they are here with us in the village, they are more accessible at all times” Man_R6_FG12 “Thanks to them our children are treated and healed without necessarily going to the health center” Woman_R2_FG18	“Faced with these illnesses, I quickly go to the health facility because hanging around leads to complications in children. The more we see children who lose consciousness along the way or at the CSPS itself. That’s why we often leave the CHWSs in the village here and go to the dispensary” Man_R1_FG14 “If it’s not at the health facility it’s complicated so when we have health problems we come directly to the health facility “Woman_R1_FG2 “They themselves come here so it’s better for us to come to the health facility too” Woman_R6_FG2
**Organizational accessibility**	“We first go to the CHWS and if they have medicines they give them to us” Woman_R6_FG3 “They help the health workers to give us care. It’s when a child is sick and it’s late that we consult the CHWS” Woman_R3_FG5 “They are recruited as part of the fight against malaria to lend a hand to the health workers in the villages” Women_R3_FG17 “It’s really very good. They help us a lot. In the winter season when you have no financial resources you see them come to treat your children for free. Without them it would be really hard because these are periods of poverty so in case of illness you have to borrow money and then sell your animals to pay it back.” Man_R3_FG12	“No, we go to the health facility “Woman_R2_FG4 “We go to the health facility because if the information on the presence of medications at their level is not constantly renewed, people forget so they send their patients directly to the health facility otherwise in previous years, for these illnesses they are the ones we call first” Man_R4_FG7 “We don’t leave them unless they are the ones who tell us to go to the health facility because it’s a bit serious” Woman_R2_FG11
**Interpersonal communication**	“They also advise us to clean up our living environment because dirt is a source of disease” Woman_R7_FG3 “They advise us to take good care of children: wash them well and protect them from the cold, wash their hands well before and after meals” Woman_R3_FG5 “We listen and apply to the letter all the advice they give us” Woman_R3_FG6 “What satisfies me the most is the respect they give to the sick person and their family, they are not arrogant.” Man_R3_FG16 “They tell us to make children sleep under mosquito nets otherwise they can get malaria from mosquitoes” Woman_R5_FG11	“It was the CHWSs who advised us not to hang around with a sick child at home, that at the first signs of illness, we should go immediately to the health center” Woman_R7_FG15 “When we call them, they come and tell us to go quickly to the dispensary because the products they have at their disposal can no longer treat a child who already has malaria” Woman_R10_FG19 “They have a period during which they walk around to distribute medicines, so when this period exceeds, you will necessarily have to go to the CSPS for care” Man_R5_FG10
**Technical competence**	“They have cough products. I send them to the CHWS who does the RDTs and if it is positive they give the medications like amoxicillin and if the illness persists they refer you to the health workers.” Woman_R2_FG1 “When your child is sick and you go to see him they treat him and give him medication” Woman_R3_FG3 “With them we can have medicines and it saves us time because we no longer need to go to the dispensary” Woman_R3_FG5 “During the rainy season, if a child is sick, we go to see them so that they can do the screening test to find out what he is suffering from before going to the clinic” Hommes_R3_FG20	“They often come to see the child and since they don’t have the medication they refer us to the CSPS” Woman_R4_FG9 “Their activities are periodic. There are times when they have the medication and when your child is sick they give it to him but at this time they don’t have any so when you have a sick person you have to go to the health facility quickly” Man_R3_FG12 “The CHWSs don’t have enough medication to treat children at home. That’s why we quickly go to the dispensary” Man_R2_FG16.

Some claim to resort to CHWs as soon as their child shows symptoms. However, this pattern did not seem to suit some households who described this recourse as a waste of time and who claimed to go directly to the health facility. The argument given in the responses was sometimes linked to the advice of some CHWs who themselves proposed this direct recourse to the health facilities and the time that can be wasted by going to CHWs first (Table 2).

#### Proximity and cultural convergence with the CHWs charged with providing iCCM.

The respondents’ identified CHWs with subsidiary terminologies most often indicating geographical and cultural proximity. The majority of households found that collaboration with CHWs was good and the conditions for using their services were satisfactory. They described a communication environment that is made up of advice and guidance. The inappropriate advice given by some CHWs was sometimes not in favor of maintaining the organizational pattern of using their services before going to the health facilities. The responses from some households showed confusion between the preventive care provided during the rainy season and the curative package that is iCCM (Table 2).

#### The dimension of technical competence.

The majority of households were convinced of the capacity of the CHWs. They described a number of stages in the administration of care provided by the CHWs such as the search for symptoms when they had recourse to them (search for symptoms, carrying out rapid diagnostic tests when the child presents symptoms, etc.), while also giving their perception. However, as shown by the verbatim statements expressing unsatisfactory quality, some households expressed doubts about the ability of the latter to resolve their problem and indicated that they preferred to go directly to health facilities in the event of fever in their children (Table 2).

### 3.3. Factors in satisfaction with the care process provided by CHWs

Using the components of satisfaction according to the conceptual model of the study, we were able to obtain the model using the ‘enter’ method and explored the factors that determine satisfaction with the quality of the process of care provided by the CHWs within the framework of the iCCM. All the conditions of use were well verified beforehand (S2 Table), and the probability of X2 (p-value significant at 0.008 (<0.05)) shows that our regression model fits well. Binary logistic regression provided results indicating that the ease of contacting an CHWs in case of need, the effective schooling of the respondent and satisfactory courtesy when administering care to your child contributed significantly to the prediction of household satisfaction with the conditions of community-based curative care of childhood diseases by s CHWs in the health districts of Boussé and Boussouma (Table 3).

**Table 3:** Satisfaction factors with the care process provided by CHWs in Boussé and Boussouma.

**Equation variables**	B	ddl	Sig.	Exp(B)	95% confidence interval for EXP(B)	
					Lower	Superior
District (1)	16,239	1	,997	11289294,180	,000	.
Municipality		6	,641			
Municipality(1)	1,248	1	1,000	3,483	,000	.
Municipality(2)	-1,252	1	,077	,286	,071	1,148
Municipality(3)	-,319	1	,715	,727	,132	4,015
Municipality(4)	-16,694	1	,997	,000	,000	.
Municipality(5)	-17,020	1	,997	,000	,000	.
Municipality(6)	-16,472	1	,997	,000	,000	.
**Easy to contact an CHWs in case of need (1)**	**-2,098**	**1**	**,048**	**,123**	**,015**	**,983**
Education level		5	,381			
Level of education(1)	2,786	1	,155	16,223	,349	753,739
**Level of education(2)**	**3,493**	**1**	**,039**	**32,882**	**1,201**	**900,345**
Level of education(3)	20,439	1	,997	752561522,043	,000	.
**Level of education(4)**	**3,465**	**1**	**,041**	**31,974**	**1,146**	**892,198**
**Level of education(5)**	**4,449**	**1**	**,028**	**85,544**	**1,612**	**4540,008**
Profession		5	,986			
Profession(1)	21,470	1	,999	2109407314,689	,000	.
Profession(2)	17,680	1	,999	47696507,420	,000	.
Profession(3)	-,494	1	,461	,610	,164	2,272
Profession(4)	-,019	1	,975	,981	,308	3,125
Profession(5)	18,028	1	,998	67503046,114	,000	.
Knowledge of common diseases_children under 5/Malaria(1)	,027	1	,968	1,027	,273	3,864
Knowledge of common diseases_children under 5/IRA(1)	-,601	1	,216	,548	,211	1,421
Knowledge of common diseases_children under 5/Diarrhea(1)	-,389	1	,419	,678	,264	1,739
Cultural accessibility (cultural assimilation of household care administration site or CHWs home)		3	,528			
Cultural accessibility (cultural assimilation of household care administration site or CHWs home)(1)	-1,131	1	,165	,323	,065	1,594
Cultural accessibility (cultural assimilation of household care administration site or CHWs home (2)	-,363	1	,826	,696	,027	17,665
cultural assimilation of care administration site household or CHWs home (3)	-,719	1	,447	,487	,076	3,116
Courtesy when treating your child (malaria, diarrhea and pneumonia)		3	,092			
**Courtesy when treating your child (treatment of malaria, diarrhea and pneumonia)(1)**	**1,670**	**1**	**,013**	**5,313**	**1,425**	**19,806**
Courtesy when treating your child (treatment of malaria, diarrhea and pneumonia)(2)	,339	1	,768	1,404	,148	13,308
Courtesy when treating your child (treatment of malaria, diarrhea and pneumonia)(3)	1,098	1	,198	2,998	,564	15,925
Awareness-raising on caring for your child (treatment of malaria, diarrhea and pneumonia)		3	,318			
Awareness-raising on caring for your child (treatment of malaria, diarrhea and pneumonia)(1)	-1,786	1	,112	,168	,019	1,516
Awareness-raising on caring for your child (treatment of malaria, diarrhea and pneumonia)(2)	-,244	1	,893	,783	,023	27,215
Awareness-raising on caring for your child (treatment of malaria, diarrhea and pneumonia)(3)	-1,371	1	,279	,254	,021	3,043
Constant	2,117	1	,301	8,307		

**a.** Introduction of variables in step 1: District, Municipality, Ease of contacting an CHWS in case of need?, Level of education, Profession, Knowledge of common diseases_children under 5 years/Malaria, Knowledge of common diseases_children under 5 years/ARI, Knowledge of common diseases_children under 5 years/Diarrhea, Cultural accessibility (speaks the same language/same habits, …), Courtesy when administering care to your child (treatment of malaria, diarrhea and pneumonia), Awareness Practical advice when administering care to your child (treatment of malaria, diarrhea and pneumonia).

## 4. Discussions

This study allowed us to note that CHWs are well known to households who describe them as community members recruited to care for them. The majority of them reassured that they trusted the care they offered and used this offer as a first resort in a certain proportion. However, the quality of communication and the doubts of some households about their ability to manage children’s health problems led to the persistence of direct recourse to health facilities. The binary logistic regression carried out to assess household satisfaction on the quality of the iCCM process allowed us to know that the ease of contacting the CHWs, the level of education and courtesy during care were significantly associated with it.

### 4.1. Availability of services and use by households

Households confirmed that they benefit from the management of fevers and other symptoms by CHWSs in the health districts of Boussé and Boussouma. Some households said they used it while others explained that they maintained direct recourse to health facilities. These problems of using the services offered by community agents had already been mentioned by Thomas Druetz et al. [10] in their study on the use of CHWs in the health districts of Kaya and Zorgho. The lack of information on this community care offer, the very preference for care offered at the level of health facilities and problems of availability of medicines had been the main reasons for the low use of CHWs. Since 2016, the Burkinabe government has been implementing a policy of free care which aims to facilitate access to services for mothers and children, initially reserved for services offered in health structures and later extended to community care since 2018 including the iCCM [24]. The results obtained show that this policy has not made it possible to resolve these pressing issues of stock-outs of medicines and consumables necessary for care mentioned by several studies [16,26,27]. The availability of the offer is variously assessed according to the results of the studies. Some studies have found coverage weaknesses with Musoke et al. [5] who reported only a proportion of 6.5% (±7.3) (n = 129) of people involved in iCCM who actually treated children for malaria, diarrhea and pneumonia during their work. Others, however, such as Geta et al. [28] found more appreciable performances with a level of implementation of iCCM of 81.5%, the availability of care of 84.2% and an acceptability of 75.3% (n = 484). For the use of these services provided by approximately 33,000 CHWs recruited by the Burkinabe government in 2016 [26–29] and in 2023 [30], residence in the villages is a priori a favorable factor. The results obtained indicate a need to properly adjust communication between CHWs and households on the quality of the offer and to work to maintain it. In light of these results, we can see the need to analyze the quality of coverage at the level of villages with particular demographics as suggested by Fletcher Njororai et al. [31] who proposed that the system be improved for villages whose concessions are dispersed, which applies perfectly in the context of Burkina Faso with villages with sometimes very dispersed concessions and also having large hamlets of very distant agricultural crops. A discrimination can be made regarding the number of CHWs to be assigned per village and the means of transport provided according to this particularity.

### 4.2. Satisfaction with the conditions of care by CHWs

Participants which come from households’ satisfaction with the conditions of community care by CHWSs is influenced by the ease of contacting the CHWs, the level of education and courtesy during care. The ease of contacting the CHWs as a factor influencing household satisfaction with the management of care is of great importance. This delay in accessing this community care had been found by Ayodele S. Jegede et al. in their study as a reason for satisfaction with CHWs, which CHWs were perceived in the said study as accessible and diligent [32].

In the same vein of understanding these iCCM programs, we note the fact that the quality of services offered by CHWs is influenced by their own level of acceptability and appropriation of iCCM. Geta et al. in their work which consisted of evaluating the implementation of iCCM in Gondar in Ethiopia, had found that the factors associated with the acceptability of the program by caregivers were the level of education, the availability of health products, the time taken to arrive on the site to offer iCCM [28]. These factors identified by our study after conducting binary logistic regression complemented the results obtained through the qualitative method of the study which highlighted problems such as the choice of direct recourse to health facilities without using CHWs, problems of availability of inputs, etc. Previous studies such as that of Sonia Hamed et al. [12] stressed the need to strengthen the means intended for the implementation of community interventions to improve the coverage of underserved populations in care. All these elements must be taken into account today to make the investments of the Government of Burkina Faso in iCCM more efficient.

## 5. Conclusion

The application of iCCM in Burkina Faso is effective and known to the populations. The quality of its implementation by CHWs is a constant concern. Through this mixed study, we wanted to understand more about the quality of this care according to the opinions of households. Households pointed out problems of availability of medicines and sometimes expressed, through the speeches of the focus groups, some reservations about the capacity of CHWs to manage the problems presented by their children. The quantitative analytical component of this study identified factors that influence this satisfaction of these households. Program managers must invest in good availability of CHWs, the main facilitators of the strategy, comparing the workload assigned to them and the staff. Efforts must be made to ensure greater availability of the inputs necessary for proper implementation with probable adaptations of the free healthcare scheme at the community level so that populations truly benefit from it. This study shows that the CHW paradigm in Burkina Faso needs to evolve, with a new format that allows the main CHWs to be in charge of care, so that they can devote time to it in a context where there are many mass treatments and health promotion needs to continue with the delegation of preventive tasks. Evaluations of the efficiency of free community healthcare could provide more refined elements for better adjustments of this public policy.

## Supporting information

S1 Table Description des dimensions de qualité des soins dans la perspective du consommateur selon BENINGUISSE. (DOCX)

S2 Table Logistic regression conditions. (DOCX)

S1 Data SPSS DATABASE. (SAV)
