# Supplementary material for: Community satisfaction with the process of curative care administered by community health workers in the Boussé and Boussouma health districts in Burkina Faso
Source: PLOS Glob Public Health. 2025 May 8;5(5):e0003951. doi: 10.1371/journal.pgph.0003951 (PMC12061114; doi:10.1371/journal.pgph.0003951)
Supplement: S1 Table — (DOCX) [file pgph.0003951.s001.docx]

**S1 Table : Description des dimensions de qualité des soins dans la perspective du consommateur selon BENINGUISSE**

| Dimensions of BENEGUISSE's conceptual model | Description of the dimensions |
| --- | --- |
| Geographical accessibility | The degree to which the proximity or remoteness of health services suits users in terms of distance/time to get there |
| Organizational accessibility | The extent to which the way services are organised is suitable for users in terms of waiting times, cleanliness and comfort of the premises and the attitude of care staff towards courtesy and respect for human dignity. |
| Interpersonal communication | The carer's ability to understand and explain the patient's health problems and to involve the patient in decisions relating to care |
| Technical skills | The carer's ability to effectively manage the patient's health problems to their satisfaction |
| Care continuity | The way in which distinct elements of patient care are linked together over time and between different providers |
