# Supplementary material for: Community satisfaction with the process of curative care administered by community health workers in the Boussé and Boussouma health districts in Burkina Faso
Source: PLOS Glob Public Health. 2025 May 8;5(5):e0003951. doi: 10.1371/journal.pgph.0003951 (PMC12061114; doi:10.1371/journal.pgph.0003951)
Supplement: S2 Table — (DOCX) [file pgph.0003951.s002.docx]

**S2 Table: Logistic regression conditions**

| **Classification table^a,b^** | | | | | | | | | | | | | | |  |
| --- | --- | --- | --- | --- | --- | --- | --- | --- | --- | --- | --- | --- | --- | --- | --- |
|  | Observed | | | | | | Preview | | | | | | | |  |
|  |  |  |  |  |  |  | Satisfaction with ASBC services | | | | | | | Correct percentage |  |
|  |  |  |  |  |  |  | No | | | Yes | | | |  |  |
|  | Satisfaction with services provided by ASBCs | | | | No | | 0 | | | 31 | | | | .0 |  |
|  |  |  |  |  | Yes | | 0 | | | 929 | | | | 100.0 |  |
|  | Global percentage | | | | | |  | | |  | | | | 96.8 |  |
| **Equation variables** | | | | | | | | | | | | | | | |
|  | | | B | E.S | | Wald | | | ddl | | Sig. | | Exp(B) | | |
|  | Constant value | | 3.400 | .183 | | 346.813 | | | 1 | | .000 | | 29.968 | | |
| **Composite tests of model coefficients** | | | | | | | | | | | | | | | |
|  | | Chi-square | | | | | | ddl | | | | Sig. | | | |
|  | Pas | 51,857 | | | | | | 30 | | | | ,008 | | | |
|  | Block | 51,857 | | | | | | 30 | | | | ,008 | | | |
|  | Model | 51,857 | | | | | | 30 | | | | ,008 | | | |
